# The Role of V-Domain Ig Suppressor of T Cell Activation (VISTA) in Cancer Therapy: Lessons Learned and the Road Ahead

**DOI:** 10.3389/fimmu.2021.676181

**Published:** 2021-05-19

**Authors:** Negar Hosseinkhani, Afshin Derakhshani, Mahdi Abdoli Shadbad, Antonella Argentiero, Vito Racanelli, Tohid Kazemi, Ahad Mokhtarzadeh, Oronzo Brunetti, Nicola Silvestris, Behzad Baradaran

**Affiliations:** ^1^ Immunology Research Center, Tabriz University of Medical Sciences, Tabriz, Iran; ^2^ Student Research Committee, Tabriz University of Medical Sciences, Tabriz, Iran; ^3^ Department of Immunology, Faculty of Medicine, Tabriz University of Medical Sciences, Tabriz, Iran; ^4^ Istituto di Ricovero e Cura a Carattere Scientifico (IRCCS) Istituto Tumori “Giovanni Paolo II” of Bari, Bari, Italy; ^5^ Department of Biomedical Sciences and Human Oncology, Aldo Moro University of Bari, Bari, Italy; ^6^ Research Center for Pharmaceutical Nanotechnology, Tabriz University of Medical Sciences, Tabriz, Iran

**Keywords:** VISTA, immune checkpoints, cancer, immunotherapy, immune-resistance

## Abstract

Immune checkpoints (ICs) have pivotal roles in regulating immune responses. The inhibitory ICs in the tumor microenvironment (TME) have been implicated in the immune evasion of tumoral cells. Therefore, identifying and targeting these inhibitory ICs might be critical for eliminating tumoral cells. V-domain immunoglobulin suppressor of T cell activation (VISTA) is a novel inhibitory IC that is expressed on myeloid cells, lymphoid cells, and tumoral cells; therefore, VISTA can substantially regulate innate and adaptive anti-tumoral immune responses. Besides, growing evidence indicates that VISTA blockade can enhance the sensitivity of tumoral cells to conventional IC-based immunotherapy, e.g., cytotoxic T lymphocyte antigen 4 (CTLA-4) inhibitors. In this regard, the current study aimed to review the current evidence about the structure and expression pattern of VISTA, its role in TME, the clinicopathological significance of VISTA, and its prognostic values in various cancers. Besides, this review intended to collect the lessons from the recent pre-clinical and clinical studies and propose a strategy to overcome tumor immune-resistance states.

## Introduction

Growing evidence indicates that inhibitory/stimulatory ICs have critical roles in regulating immune responses (1). These IC axes can be established between tumor-infiltrating immune cells and tumoral cells, which have led to the development of novel immunotherapy approaches (2, 3). Indeed, a better understanding of these interactions between tumoral cells and TME might open a new era for treating cancer patients.

ICs, which can be expressed on immune cells and tumor cells, can regulate the immune responses. These molecules are essential in maintaining the body’s homeostasis; however, the expression of inhibitory ICs, e.g., programmed cell death ligand 1 (PD-L1), can shield tumoral cells from anti-tumoral immune responses. Thus, suppressing these inhibitory ICs has gained special attention in treating various cancers (4–6). Indeed, targeting these inhibitory ICs, e.g., CTLA-4 and programmed death receptor 1 (PD-1), has been associated with activated anti-tumoral immune responses in affected patients (7–9).

VISTA, also referred to as PD-1 homolog (PD-1H), differentiation of embryonic stem cells 1 (Dies1) (10), DD1α, Gi24, SISP1 (11), B7-H5 (12), and C10orf54, is a novel inhibitory IC (13). This study aimed to review the structure and expression pattern of this inhibitory IC in normal and tumoral cells. Furthermore, the review intended to highlight the role of VISTA in regulating immune responses and immune-resistant states following conventional IC-based immunotherapy in various cancers.

## The Structure of VISTA

The *VISTA* gene, also referred to as *Vsir*, has 930 base pairs that can be translated to a type I transmembrane protein in mice (14). Murine VISTA, a protein with 309 amino acids (aa), has shown homology with the B7 family members. A single extracellular Ig-V domain with 136-aa linked to a 23-aa stalk segment has 76% homology with the human protein. A transmembrane domain, which contains 21-aa and a cytoplasmic tail with 97-aa, shares up 90.6% identity and has no immunoreceptor tyrosine-based inhibitory motif (ITIM), immunoreceptor tyrosine-based activation motif (ITAM), and immunoreceptor tyrosine-switch motif (ITSM) (14). Protein kinase C binding sites and proline-rich segments have been identified in the cytoplasmic tail; however, their exact functions have not been well-known (14). Human VISTA with 311-aa contains a single extracellular IgV domain with 130-aa linked to the 33-aa stalk segment. Besides, the human VISTA includes a 20-aa transmembrane region linked to a cytoplasmic tail with 96-aa (14). Genetical analyses have shown that the *Vsir* gene is located on chromosome 10 (10q22.1) in the intron of the *CDH23* gene, distant from other B7 members. Highly conserved sequences of VISTA can be indicative of critical functions (14).

In contrast with other members of the B7 family, VISTA has a single IgV domain that contains three extra cysteine residues, i.e., Cys44, Cys83, and Cys144, and one excess cysteine, i.e., Cys177, in the stalk segment. Besides, conventional B7 members fold has nine beta-strands, but the fold of VISTA has ten beta-strands. Furthermore, it has an extracellular domain, including an extra helix chain (FQDL) instead of the normal beta-strand C′ in its positively charged patch. Moreover, VISTA has a loop between C” and D, and two additional S–S bonds that other members of the B7 family lack these disulfide bonds (15, 16). The presence of additional “H” β-strand and “clamping” disulfide in the IgV-like domain of VISTA can limit its orientation on the cell surface (17). The surface-exposed histidine cluster has the primary function in inhibiting T cells. These specific features make VISTA individual from other members of the B7 family. VISTA has been aligned by protein sequence analysis with the B7 family of ligands and receptors; the B7 family ligands, i.e., B7.1, B7.2, PD-L1, PD-L2, and ICOSL, possess Ig-V and Ig-C domains. In contrast, the CD28, PD-1, and ICOS receptors include one Ig-V domain. Although it has been shown that VISTA has ligand function, it contains one Ig-V domain like other receptors in the B7 family (14). Therefore, VISTA can serve as a ligand or receptor or both in the intercellular cross-talk.

## The Expression Level of VISTA in the Normal Cells and Tissues

The expression of murine VISTA in embryonic stem cells can affect the differentiation of stem cells *via* regulating the bone macrophage protein 4 (BMP4) signaling (18–20). Besides, it can affect the conversion of preadipocytes to adipocytes (21). Initially, *Vsir* gene expression was determined in lymphoid tissues, e.g., bone marrow, spleen, lymph node, and thymus of adult mice (22). It is now proven that its expression can be increased following inflammation in the lung and small intestine. Moreover, non-hematopoietic tissues like the heart, brain, lung, muscle, testis, ovary, and placenta have low but detectable VISTA mRNA expression (14). Within hematopoietic cells, CD11b^high^ myeloid cells, e.g., monocytes, conventional dendritic cells (DCs), macrophages, and circulating granulocytes, can overexpress VISTA; however, CD11^intermediate^ myeloid cells might have an intermediate level of VISTA expression (16). Furthermore, VISTA has essential roles in regulating the inflammatory functions of macrophages (23). Among lymphocytes, CD4^+^ T cells and FoxP3^+^ regulatory T cells (Tregs) can overexpress VISTA; however, memory CD4^+^ T cells, CD8^+^ T cells, natural killer cells have lower levels of it. Also, studies have failed to identify the surface expression of VISTA on the B cells (14).

In human tissues, recent findings have indicated that the brain, stomach, thyroid, and hematopoietic tissues overexpress human VISTA; however, the heart, liver, bone, and spleen demonstrate the lowest expression (22, 24). Throughout pregnancy, VISTA expression in the placenta might be attributable to its role in allo-fetal tolerance (25). Within hematopoietic tissues, the highest expression level of VSITA is identified in myeloid cells, e.g., CD14^dim^CD16^+^ patrolling monocytes, CD11c^low^CD123^+^HLA-DR^+^ DCs, microglia, neutrophils, CD14^+^CD16^+/−^ inflammatory monocytes, and CD11c^+^CD123^low^HLA-DR^+^ DCs. In the lymphoid tissues, the main expression of VISTA is found in naïve CD4^+^ T cells, FoxP3^+^ Tregs, and other subsets of T cells. Furthermore, VISTA in CD44^low^ CD62L^hi^ naïve T cells has a significant role in maintaining the quiescence phenotype of cells (26). Also, CD56^low^ natural killer cells demonstrate relatively low VISTA expression (24, 25). Although VISTA expression has not been identified on B cells, it has been demonstrated that plasma cells can substantially express VISTA (27).

Studies have shown that VISTA expression can be altered under inflammatory conditions. For instance, the expression of VISTA in CD14^+^ monocytes can be upregulated following the stimulation of toll-like receptors (TLRs) and secretion of interleukin (IL)-10/interferon-γ (IFN-γ) in HIV infection (24). Moreover, it has been shown that *Vsir^−−^* mice models can develop severe inflammatory responses to foreign and self-antigens (28).

## The Role of VISTA in the Regulation of Immune Responses

Accumulating evidence has indicated that VISTA can be involved in the regulation of immune responses. It has been shown that *VISTA^−/−^* mice can upregulate the proinflammatory cytokines and chemokines in multiple tissues, leading to autoimmunity development (25, 29). In line with this, *Vsir^-−/−^* mice are prone to develop auto-activated T cells and proinflammatory cytokines production, which can lead to acute hepatitis development (15). However, VISTA deficiency does not always ensure the experimental autoimmune encephalomyelitis (EAE) development, which owes to the fact that other ICs, e.g., PD-1, and CTLA-4, can also regulate immune responses. Besides, *VISTA* suppression in tumor-infiltrating lymphocytes has not remarkably led to tumor elimination (25, 30). Consistent with these, knocking out VISTA/PD-1 has been associated with the development of auto-activated T cells and chronic inflammation (31). These findings suggest that dual blockade of VISTA and PD-1 can have a synergistic effect on regulating T cells.

Recent findings have shown that VISTA can also regulate TLRs signaling in myeloid cells and control the myeloid-derived-suppressor cells (MDSCs) and DC-mediated inflammation. Indeed, blocking VISTA can lead to proinflammatory cytokines accumulation *via* the TLR/MyD88-mediated pathway in bacterial infection (32). Furthermore, the localization of VISTA in synovial membrane cells, neutrophils, and scattered cells in lymphocytes rich site can be involved in developing arthritis and VISTA deficiency can attenuate arthritis-induced joint injury (33).

Of interest, VISTA also has critical roles in allergy reactions and T helper 2-mediated immune responses. In the *Vsir^−/−^* mice, T helper 2-mediated pulmonary inflammation has led to severe asthma attacks. Besides, VISTA deficiency has led to the release of airway inflammatory cytokines, leading to disease exacerbation (34). Overall, these studies indicate the critical role of VISTA in regulating immune responses.

## VISTA as a Ligand or Receptor

Although the function of VISTA as a ligand or receptor remains to be elucidated, studies have suggested two ligands for this promising target. Using immobilized ectodomain of VISTA, which can inhibit T cell-mediated immune responses *via* interaction with inhibitory receptors on T cells and promote the development of Foxp3^+^ Tregs, has been indicative of the ligand activity of VISTA (14, 25). Moreover, VISTA expression on MCA105 tumor cells has shown that tumoral VISTA acts as a ligand, which can facilitate tumor growth (14). In line with these, VISTA expression on tumor cells and bone marrow-derived DCs can be associated with inhibited T cell proliferation in A20 B lymphoma. These findings support the idea that VISTA acts as a ligand in regulating immune responses (14).

**Figure 1:**
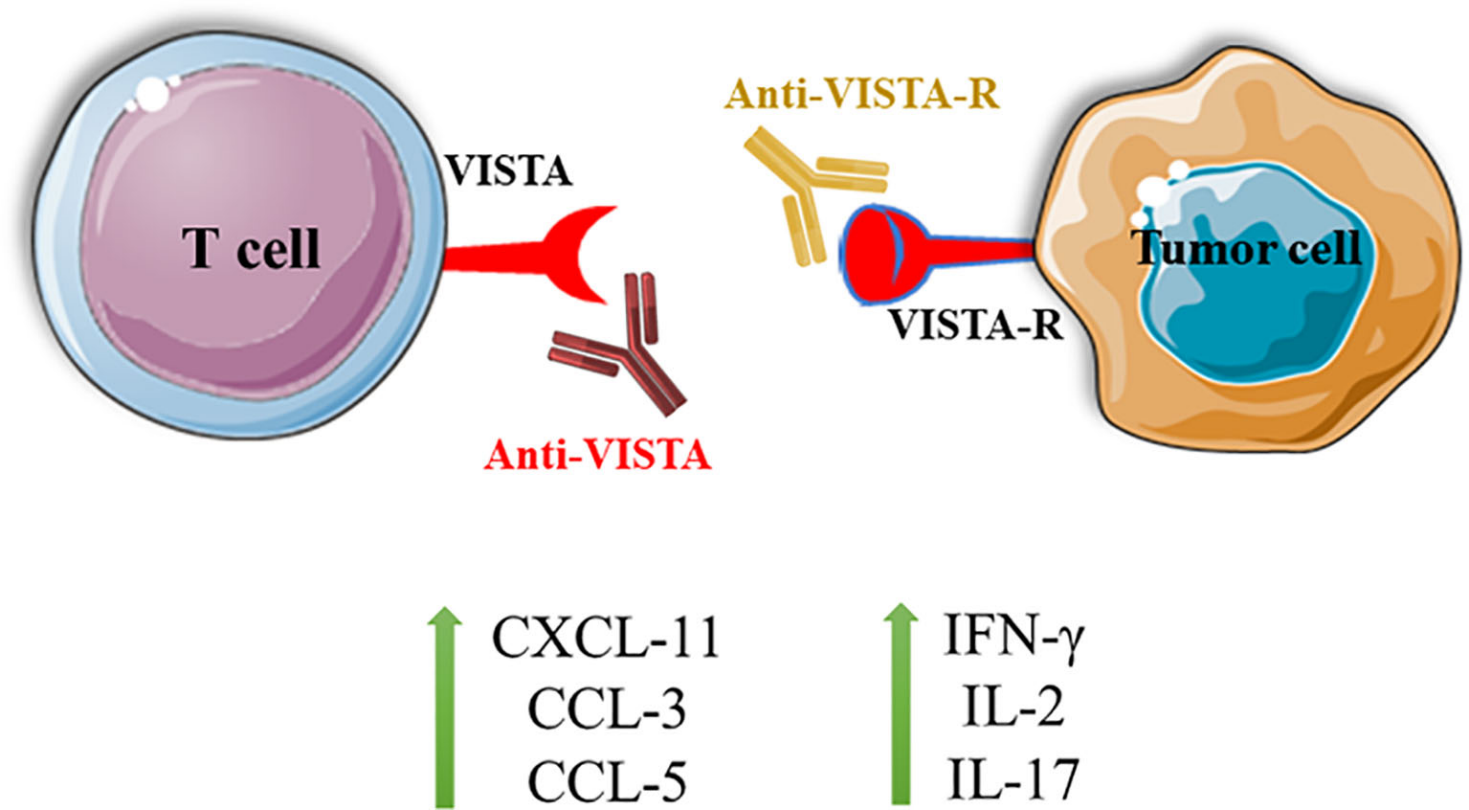
Blocking the interaction between VISTA and VISTA-R can lead to T cell activation and the upregulation of IFN-γ, IL-2, IL-17, CXCL-11, CCL-3, and CCL-5. VISTA, V-domain Ig suppressor of T cell activation; VISTA-R, VISTA-receptor.

However, Johnston et al. have shown that VISTA has a unique structure in the extracellular domain, which is enriched with histidine residues. As a side chain of histidine, imidazole can become deprotonated in low pH so that VISTA can bind to its receptor in the acidic environment of TME. P-selectin glycoprotein ligand-1 (PSGL-1) is a pH-dependent ligand that can bind to VISTA following its post-translational modifications. Besides, VISTA and PSGL-1 have similar expression patterns on leukocytes, indicating the possibility of their interaction. Korman et al. have shown that pH-selective antibodies for targeting VISTA can suppress the interaction between VISTA and PSGL-1 (12). V-set and immunoglobulin domain containing 3 (VSIG-3) is another proposed ligand for VISTA. Wang et al. have shown that VSIG-3/immunoglobulin superfamily member 11 (IGSF11) can demonstrate remarkable interactions with VISTA. Indeed, blocking VISTA by antibodies has been associated with decreased VISTA interaction with VSIG-3, leading to the upregulation of IFN-γ, IL-2, IL-17, CCL-5, CCL-3, and CXCL11 (10) (**Figure 1**). More studies about the exact ligands or receptors of VISTA can be a critical step in successfully targeting this novel IC for cancer immunotherapy.

## VISTA in TME

TME comprises tumor cells, immune cells, blood vessels, fibroblasts, extracellular matrix, and signaling molecules. The cross-talk between tumoral and immune cells in TME has essential roles in determining the fate of tumoral cells (35–37). Although inhibitory ICs, e.g., CTLA-4, PD-1, T cell immunoglobulin and mucin-domain containing-3 (TIM-3), lymphocyte activation gene-3 (LAG-3), and VISTA, can maintain the body’s homeostasis and prevent autoimmunity, tumoral cells can also express these inhibitory ICs and shield themselves from anti-tumoral immune responses (38, 39). Consistent with this, it has been shown that tumor-infiltrating immune cells, e.g., CD11b^+^Gr1^+^ myeloid cells and FoxP3^+^ Tregs, can overexpress VISTA and attenuate anti-tumoral immune responses. Also, the hypoxic condition of TME can lead to VISTA overexpression, resulting in the immune evasion of tumoral cells (40, 41). In line with this, Nowak et al. have demonstrated that blocking VISTA can inhibit the recruitment of MDSCs and tumor-infiltrating lymphocytes (42). Besides, Wang et al. have shown that VISTA upregulation can lead to tumor growth in mice bearing fibrosarcoma (14). Indeed, VISTA can be substantially overexpressed in the tumor-infiltrating immune cells of various cancers, e.g., melanoma (43, 44), gastric cancer (19), prostate cancer (45), colorectal cancer (46), and acute myeloid leukemia (47). Besides, recent findings have shown that VISTA can also be expressed on tumoral cells, e.g., hepatocellular (48), endometrial cancer (49), ovarian cancer (49), gastric cancer (50), and non-small cell lung cancer (51). Since VISTA expression on tumor cells can lead to tumor development and it signaling pathway is different from the signaling pathways of other inhibitory ICs such as PD-L1, dual blockade of tumoral VISTA/PD-L1 can lead to the synergic inhibitory effect on tumor development (41).

## The Role of VISTA in the Different Cancers

Recent findings have demonstrated that VISTA is overexpressed in cholangiocarcinoma, glioblastoma multiforme, renal clear cell carcinoma, acute myeloid leukemia, lower-grade glioma, and pancreatic adenocarcinoma compared to healthy tissue. However, bladder cancer, invasive breast carcinoma, cervical squamous cell carcinoma, endocervical adenocarcinoma, colorectal cancer, diffuse large B cell lymphoma, kidney chromophobe, lung adenocarcinoma, lung squamous cell carcinoma, prostate cancer, melanoma, endometrial carcinoma, and uterine carcinosarcoma have a lower level of VISTA expression (52). The following sections aim to discuss the role of VISTA in the TME, the cross-talk between VISTA and other ICs, its clinicopathological significance, and its prognostic value in various cancers.

### Melanoma

Recent findings have shown that VISTA can be expressed on various cells of TME, e.g., keratinocytes, fibroblasts, and cancer-associated fibroblast-like cells (53, 54). Kakavand et al. have demonstrated that anti-PD-1/anti-CTLA-4 therapy can upregulate VISTA expression, leading to immune-resistance development and tumor relapse (43). Besides, the VISTA expression has been associated with Foxp3^+^ Tregs (43), CD68^+^ infiltrating macrophages (55), and PD-1^+^ inflammatory cells. Moreover, VISTA expression has been associated with the inferior disease-specific survival of affected patients (44). In cutaneous melanoma, VISTA and CD33 are positively correlated, and these markers have been associated with the advanced tumor stage (56). In mice bearing B16-BL6 cells, blocking VISTA has been associated with decreased recruitment of MDSCs and myeloid DCs, resulting in attenuating anti-tumoral immune responses (32). Besides, Rosenbaum et al. have reported that VISTA expression is associated with tumor growth in the early phases, indicating that VISTA has an essential role in tumor growth (57).

Forkhead box D3 (FOXD3) is a transcription factor that affects the therapy resistance in melanoma (58). Recent data have indicated that FOXD3 can bind to the intron 1 of the *Vsir* gene and regulates VISTA expression at the transcript level (57). Further investigations are needed to elucidate the role of this regulatory cross-talk between VISTA and FOXD3 in melanoma biology.

### Gastrointestinal Cancers

Recent findings have indicated that tumor-infiltrative immune cells have pivotal roles in determining the response rate of patients with gastrointestinal cancers (59–61). Although myofibroblasts can overexpress VISTA in gastric cancer, promotor methylation and *miR-125a-5p* expression can substantially regulate VISTA in gastric cancer (19). Böger et al. have reported that VISTA cannot serve as a prognostic factor for gastric cancer patients. However, a significant association between VISTA and PD-L1 expression might support the dual blockade of VISTA/PD-L1 strategy for gastric cancer patients (50). In line with this, the dual blockade of VISTA/PD-L1 has elicited synergic effects in terms of enhancing anti-tumoral immune responses in mice bearing tumors (41). Although VISTA is not overexpressed in pancreatic cancer cells (62), Liu et al. have reported that VISTA is overexpressed on the tumor-infiltrating immune cells of pancreatic cancer (63). Blando et al. have also shown that CD68^+^ macrophages can overexpress VISTA in metastatic pancreatic ductal adenocarcinoma, and VISTA blockade can restore anti-tumoral immune responses in pancreatic cancer (55).

Growing evidence indicates that *Vsir* expression is upregulated in colorectal cancer cells, especially in microsatellite instability-high (MSI+) colorectal cancer cells (46, 64). The protein expression of VISTA has been associated with immunosuppressive TME and the expression of other inhibitory ICs (46). Furthermore, the upregulated mRNA expression of VISTA in peripheral blood mononuclear cells (PBMCs) of colorectal cancer patients can indicate VISTA’s role in inhibiting anti-tumoral immune responses (65). Besides, Kitsou et al. have demonstrated that VISTA expression is positively associated with tumor-infiltrating lymphocytes in colon adenocarcinoma (66). Zong et al. have indicated that VISTA upregulation is associated with improved prognosis, mismatch repair deficiency, and early tumor stage (67). Hummingbird is a VISTA-targeting agent that administration of it has been associated with increased IL-2, IL-17, and IFN-γ expression in mice bearing colorectal cancer (68). Zhang et al. have highlighted a strong positive correlation between VISTA expression with advanced tumor grade and increased CD8^+^ T cells infiltration in hepatocellular carcinoma. Indeed, the overexpression of VISTA on tumor cells and VISTA^+^ CD8^+^ T cells have been associated with improved prognosis in affected patients (48).

### Ovarian Cancer, Endometrial Cancer, and Gestational Trophoblastic Neoplasia

In ovarian cancer, VISTA can be expressed in endothelial cells, tumor-infiltrating cells, and tumoral cells. Moreover, VISTA is upregulated in choriocarcinoma and placental site trophoblastic tumor (69). VISTA overexpression in ovarian cancer cells and its association with the advanced tumor stage might indicate the critical roles of VISTA in ovarian cancer development. However, its expression level might not serve as a valuable prognostic marker for ovarian cancer patients (49). In ovarian and endometrial cancer, VISTA has been implicated in suppressing effector T cell activation and downregulating proinflammatory cytokines (13). Recent findings have indicated a strong association between VISTA expression in tumor-infiltrating cells with PD-L1 in high-grade serous ovarian cancer. VISTA expression has been associated with a favorable prognosis in patients with high-grade serous ovarian cancer (70).

### Non-Small Cell Lung Cancer

In non-small cell lung cancer, VISTA can be upregulated in immune cells, e.g., CD68^+^ macrophages, CD8^+^ T cells, and CD4^+^ T helper cells. VISTA expression has been associated with the expression of PD-1 and PD-L1 in non-small cell lung cancer patients. Besides, there has been a remarkable association between VISTA expression with EGFR mutation and improved 5-year overall survival (51). In line with this, Brcic et al. have shown that VISTA can be overexpressed in the lymphocytes in squamous cells and adenocarcinoma of the lung (71). Furthermore, high VISTA/PSGL1 co-localization has been associated with improved overall survival in non-small cell lung cancer patients (72).

### Renal Cell Carcinoma

Hong et al. have shown that *C10orf54* expression can be upregulated in clear cell renal cell carcinoma compared to adjacent normal cells, and its expression level can be higher than the level of PD-L1 expression. Also, its expression is associated with inhibited CD8^+^ T cell activation (73). Consistent with this, another study has highlighted upregulated gene expression of VISTA in clear cell renal cell carcinoma cells (74). In clear cell renal cell carcinoma, VISTA is mainly upregulated in CD14^+^HLA-DR^+^ macrophages, and its blockade has been associated with decreased tumor growth (73).

### Breast Cancer

Zong et al. have shown that VISTA can be expressed in immune and tumoral cells in invasive ductal breast cancer. Indeed, VISTA can be overexpressed in immune cells of patients with estrogen receptor-negative, progesterone receptor-negative, human epidermal growth factor receptor 2 (HER2)-positive, triple-negative, and basal-like breast cancers. Besides, its expression has been associated with improved relapse-free survival and disease-specific survival in patients with estrogen receptor-negative, progesterone receptor-negative, and basal-like invasive ductal breast cancers (75). Consistent with these, Xie et al. have demonstrated that VISTA can be overexpressed in CD68^+^ macrophages, CD4^+^ T cells, CD8^+^ T cells, and CD20^+^ B cells. Also, VISTA expression has been positively associated with higher tumor grade, lymph node involvement, and PD-1 overexpression (76). The positive correlation between VISTA and PD-1 might support the VISTA/PD-1 dual blockade for treating breast cancer patients (75).

### Oral Squamous Cell Carcinoma

In oral squamous cell carcinoma, VISTA overexpression has been associated with lymph node metastasis and the inferior overall survival of affected patients. Besides, its expression has been associated with PD-L1, CTLA-4, IL-13 receptor α2, phosphoinositide 3-kinases (PI3K), p-STAT3, CD11b, and CD33 (77). Although VISTA blockade has been associated with the stimulation of CD8^+^ T cells, its blockade has not inhibited Treg recruitment. Indeed, the VISTA/CTLA-4 dual blockade has substantially inhibited tumor growth in squamous cell carcinoma (40).

### Glioblastoma

In the central nervous system, VISTA is mainly expressed in parenchyma resident microglia and macrophages (78). Since microglia can express inhibitory ICs, they have been implicated in glioblastoma development. Consistent with this, Flies et al. have shown that VISTA can inhibit the proliferation of CD4^+^ T cells and antigen-presenting cells. Furthermore, they have demonstrated that VISTA^knocked-out^ mice are more resistant to develop glioma compared to wild-type mice (15).

### Acute Myeloid Leukemia

VISTA is mainly expressed in the MDSCs of patients with acute myeloid leukemia. Besides, leukemic blasts can overexpress VISTA (79, 80). VISTA blockade in MDSCs has been associated with increased proliferation of CD8^+^ T cells. Furthermore, positive associations between VISTA and PD-1 in CD8^+^ T cells, T helper cells, and Tregs can indicate the synergistic effects of the dual VISTA/PD-1 blockade strategy (47).

## VISTA as a Prognostic Factor in Cancers

In high-grade serous ovarian cancer, non-small cell lung cancer, hepatocellular carcinoma, and pT1/T2 stage esophageal cancer, VISTA expression has been associated with improved overall survival of affected patients (48, 51, 70, 81). However, VISTA overexpression has been associated with the inferior overall survival of patients with oral squamous cell carcinoma (77). A recent meta-analysis has shown that VISTA overexpression is associated with improved overall survival and increased tumor-infiltrating cytotoxic T lymphocytes in cancer patients (82).

## VISTA and Cancer Therapy

**Figure 2:**
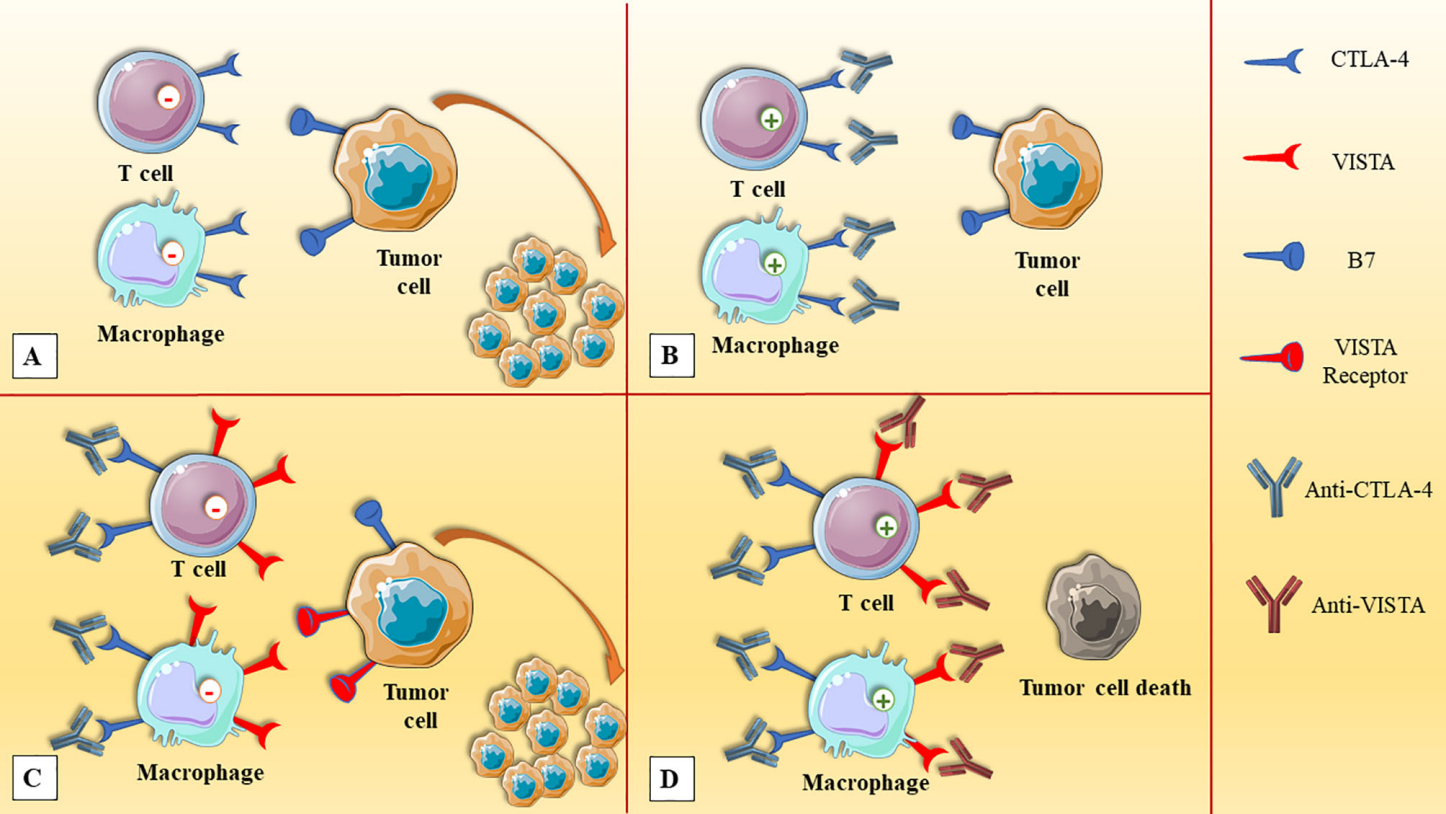
The upregulation of VISTA in prostate cancer after anti-CTLA-4 therapy. **(A)** The expression of CTLA-4 on immune cells, e.g., T cells and macrophages, and its interaction with tumoral cells can attenuate anti-tumoral immune responses. **(B)** The administration of anti-CTLA-4 can stimulate anti-tumoral immune responses. **(C)** After anti-CTLA-4 therapy, the upregulation of VISTA inhibits immune cell activation and leads to tumor expansion. **(D)** Dual VISTA/CTLA-4 blockade can elicit synergistic responses and leads to the elimination of prostate cancer cells. CTLA-4, cytotoxic T-lymphocyte-associated protein-4; VISTA, V-domain Ig suppressor of T cell activation.

Since the signaling pathways of VISTA are different from other inhibitory ICs, e.g., PD-1 and CTLA-4, the combined blockade of inhibitory ICs can elicit synergistic effects in terms of stimulating anti-tumoral immune responses. Furthermore, patients who have acquired resistance to PD-1/CTLA-4-targeting antibodies might benefit from VISTA blockade (41). Gao et al. have demonstrated that VISTA and PD-L1 are upregulated following treatment with ipilimumab, which is a CTLA-4-targeting monoclonal antibody. Indeed, VISTA overexpression in tumor-infiltrating lymphocytes and CD68^+^ macrophages cells following ipilimumab therapy might be the reason for the low response rate of this monotherapy in patients with prostate cancer (45) (**Figure 2**). SG7, a VISTA-targeting monoclonal antibody, can suppress the interaction between VISTA and VSIG3 or PSGL-1, and also, the combined therapy of SG-7 with PD-1-targeting monoclonal antibodies has shown superiority over monotherapies in colon adenocarcinoma (83). Moreover, the combination of inhibitory ICs inhibitors with other cancer therapies might be another appealing strategy to increase the response rates of cancer therapies. Pilones et al. have reported that focal radiotherapy and cyclophosphamide can increase the response rates to VISTA inhibitors and PD-1 inhibitors in mice bearing triple-negative breast cancers (84).

## VISTA in Clinical Trials

**Figure 3:**
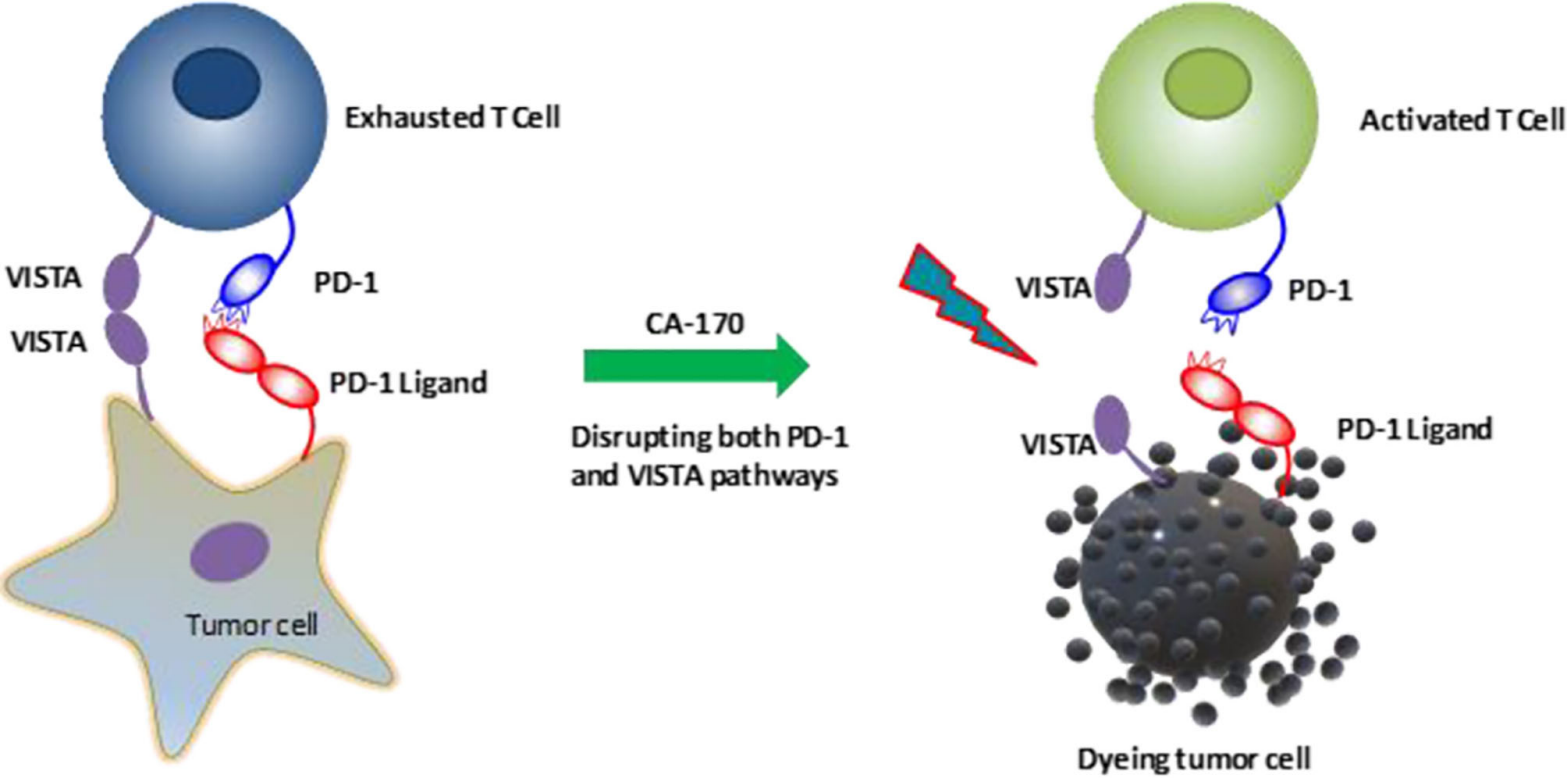
CA-170, an orally administered agent, can directly block the interaction of PD-1/PD-L1 and VISTA/VISTA-receptor and reactivate T cells (85). VISTA, V-domain Ig suppressor of T cell activation, PD-1, Programmed cell death protein-1; PD-L1, Programmed death-ligand 1.

**Table 1** aims to summarize the clinical trials evaluating the efficacy and safety of VISTA blockade in various cancers. CA-170, an orally administered agent, can directly target PD-L1, PD-L2, and VISTA that lead to IFN-γ release (**Figure 3**). Phase I of this agent was tested on patients with solid tumors or lymphoma (NCT02812875). Besides, another clinical trial evaluates its efficacy in patients with lung cancer, Hodgkin lymphoma, head and neck/oral cavity cancer, and MSI-high cancer (CTRI/2017/12/011026).

**Table 1:** VISTA in clinical trials.

Medication	Cancer type	Status	Clinical trial phase	Location	Identifiers
**CA-170**	Advanced solid tumors or lymphomas	Completed	Phase I	USA	NCT02812875
**CA-170**	lung cancer, head/neck/oral cavity cancer, MSI-H positive cancers, and Hodgkin lymphoma	Terminated	Phase II	India	CTRI/2017/12/011026
**JNJ-61610588**	Advanced tumors	Terminated	Phase I	USA	NCT02671955
**CI-8993**	Relapsed/refractory solid tumors	Recruiting	Phase I	USA	NCT04475523

MSI-H, microsatellite instability-high.

JNJ-61610588 is the first fully-humanized monoclonal IgG1 antibody that targets VISTA. In 2016 this agent was investigated in a clinical trial (NCT02671955). This clinical trial was designed to determine its safety and efficacy in patients with lung, pancreatic, cervical, colorectal, and head and neck cancers. However, this study was terminated in 2018 because of financial issues. CI-8993, a novel fully human IgG1 monoclonal antibody against VISTA that will be evaluated in patients with solid tumors (NCT04475523). Blocking VISTA ligands can also stimulate anti-tumoral immune responses. Verseau Therapeutics, Inc. has developed a monoclonal antibody against PSGL-1 (VTX-0811), which can promote inflammatory responses by reprogramming tumor-associated macrophages (27).

## Conclusion

The immunosuppressive TME, which inhibitory ICs have been implicated in its development, can substantially attenuate anti-tumoral immune responses and pave the way for tumor development. Therefore, identifying and targeting these inhibitory ICs can stimulate anti-tumoral immune responses and lead to tumor elimination. As a novel inhibitory IC, VISTA can be overexpressed on myeloid lineages, lymphoid lineages, tumor-infiltrating immune cells, and tumor cells; thus, it can substantially inhibit anti-tumoral immune responses. Based on the current evidence, the combined blockade of inhibitory ICs, e.g., VISTA/PD-1 blockade, can increase the response rates of affected patients to cancer therapies. However, the safety and tolerability of this approach should be evaluated in clinical trials before its translation into the clinics.

## Author Contributions

NH collected data and wrote the initial version of the paper. AD and MS revised the manuscript. AA, VR, TK, AM, and OB, contributed to English editing. BB and NS supervised the manuscript. All authors contributed to the article and approved the submitted version.

## Funding

This study was supported by the Immunology Research Center of Tabriz University of Medical Sciences, Tabriz, Iran (grant number 65671).

## Conflict of Interest

The authors declare that the research was conducted in the absence of any commercial or financial relationships that could be construed as a potential conflict of interest.
